# Evaluation of Retinal Blood Flow in Patients with Monoclonal Gammopathy Using OCT Angiography

**DOI:** 10.3390/jcm12165227

**Published:** 2023-08-11

**Authors:** Cecilia Czakó, Dóra Gerencsér, Kitti Kormányos, Klaudia Kéki-Kovács, Orsolya Németh, Gábor Tóth, Gábor László Sándor, Anita Csorba, Achim Langenbucher, Zoltán Zsolt Nagy, Gergely Varga, László Gopcsa, Gábor Mikala, Illés Kovács, Nóra Szentmáry

**Affiliations:** 1 Department of Ophthalmology, Semmelweis University, 1085 Budapest, Hungary; 2 Department of Ophthalmology, Markusovszky University Teaching Hospital, 9700 Szombathely, Hungary; 3 Experimental Ophthalmology, Saarland University, 66424 Homburg, Germany; 4 3rd Department of Internal Medicine and Haematology, Semmelweis University, 1085 Budapest, Hungary; 5 Department of Haematology and Stem Cell-Transplantation, South-Pest Central Hospital-National Institute for Hematology and Infectious Diseases, 1097 Budapest, Hungary; 6 Department of Ophthalmology, Weill Cornell Medical College, New York City, NY 10065, USA; 7 Dr. Rolf M. Schwiete Center for Limbal Stem Cell and Congenital Aniridia Research, Saarland University, 66424 Homburg, Germany

**Keywords:** monoclonal gammopathy, multiple myeloma, hyperviscosity syndrome, optical coherence tomography angiography

## Abstract

Background: Monoclonal gammopathy (MG) is characterized by monoclonal protein overproduction, potentially leading to the development of hyperviscosity syndrome. Objective: To assess retinal circulation using optical coherence tomography angiography (OCTA) parameters in patients with monoclonal gammopathy. Methods: OCTA measurements were performed using the Optovue AngioVue system by examining 44 eyes of 27 patients with MG and 62 eyes of 36 control subjects. Superficial and deep retinal capillary vessel density (VD SVP and DVP) in the whole 3 × 3 mm macular and parafoveal area, foveal avascular zone (FAZ) area, and central retinal thickness (CRT) were measured using the AngioAnalytics software. The OCTA parameters were evaluated in both groups using a multivariate regression model, after controlling for the effect of imaging quality (SQ). Results: There was no significant difference in age between the subjects with monoclonal gammopathy and the controls (63.59 ± 9.33 vs. 58.01 ± 11.46 years; *p* > 0.05). Taking into account the effect of image quality, the VD SVP was significantly lower in the MG group compared to the control group (44.54 ± 3.22% vs. 46.62 ± 2.84%; *p* < 0.05). No significant differences were found between the two groups regarding the other OCTA parameters (*p* > 0.05). Conclusions: A decreased superficial retinal capillary vessel density measured using OCTA in patients with MG suggests a slow blood flow, reduced capillary circulation, and consequent tissue hypoperfusion. An evaluation of retinal circulation using OCTA in cases of monoclonal gammopathy may be a sensitive method for the non-invasive detection and follow-up of early microcirculatory dysfunction caused by increased viscosity.

## 1. Introduction

Monoclonal gammopathies are characterized by the proliferation of clonal plasma cells, resulting in the presence of serum M-protein, also known as paraproteinemia [1]. The most common form of the disease is monoclonal gammopathy of undetermined significance (MGUS). MGUS occurs in 3% of individuals aged 50 or older and 5% of those 70 years of age or older [2]. According to the International Myeloma Working Group (IMWG), MGUS is characterized by the presence of serum M-protein less than 3 g/dL, monoclonal plasma cells less than 10% in the bone marrow, and the absence of end-organ damage associated with plasma cell proliferative disorder—such as bone lesions, anemia, hypercalcemia, and renal insufficiency [3]. The most common type of M-protein is IgG (69%), followed by IgM (17%), IgA (11%), or biclonal (3%). Patients with IgG and IgA monoclonal gammopathy tend to progress to multiple myeloma (MM) and rarely, to light chain (AL) amyloidosis. Nevertheless, those with IgM monoclonal immunoglobulin overproduction may progress to Non-Hodgkin’s lymphoma, Waldenström’s macroglobulinemia (WM), chronic lymphocytic leukemia, and AL amyloidosis. MGUS is considered to be a premalignant condition, as the risk of progression to MM is 1% per year for patients with IgG MGUS and the risk of progression to WM is 1.5% for those with IgM MGUS. The cumulative risk of progression is 10% at 10 years, 18% at 20 years, and 36% at 40 years. Associations between MGUS and other conditions such as renal disease, polyneuropathy, and also ocular conditions were recently described, where the presence of the M-protein may contribute to the development of organ dysfunctions [4].

Ocular manifestations of monoclonal gammopathy are not common and mainly affect the anterior segment. Ophthalmic disorders are due to the deposition of monoclonal kappa light chain immunoglobulins in the ocular tissues or hyperviscosity syndrome from increased circulating serum immunoglobulins. Proptosis, conjunctival and corneal crystalline deposits, copper deposition in Descemet’s membrane, maculopathy with serous macular detachment, autoimmune retinopathy, central retinal vein occlusion, and hyperviscosity-related retinopathy have been described in the literature [5,6,7,8,9,10]. Crystalline keratopathy may be present as corneal deposits of immunoglobulin light chains in less than 1% of patients with monoclonal gammopathy [11]. Retinal vascular manifestations include venous dilatation, retinal hemorrhages, cotton wool spots, and microaneurysms. These findings have been attributed to hyperviscosity due to elevated levels of circulating immunoglobulins, which is most frequently observed in Waldenström’s macroglobulinemia, where the large molecular weight serum IgM pentamer leads to aggregation and a significant increase in serum viscosity [12]. Ocular symptoms may be the initial presentation of monoclonal gammopathy; as a result, earlier detection may allow closer monitoring for its progression to multiple myeloma or Waldenström’s macroglobulinemia.

Optical coherence tomography angiography (OCTA) is increasingly becoming a promising and important imaging technique in ophthalmology due to its ability to non-invasively visualize the different retinal and choroidal vascular layers. Instead of intravenous dye injection, OCTA uses motion contrast technology to image the blood flow by detecting the movement of red blood cells. OCTA is a useful imaging modality for evaluating ophthalmological diseases such as diabetic retinopathy, age-related macular degeneration, retinal artery and vein occlusions, and glaucoma. Moreover, besides a visualization of the retinal and choroidal vasculature, OCTA provides numerous data on retinal blood flow; thus, it is highly suitable for objective follow-ups on different diseases [13].

Due to the less frequent involvement of the posterior segment in monoclonal gammopathy, only a few papers have been published assessing retinal circulation using OCT angiography [14,15,16]. As retinal blood flow may reflect the systemic blood circulation, an easy method for recognizing even mild circulatory changes can have many benefits, such as early diagnosis and the detection of progression, the possibility of a closer follow-up of these patients, and monitoring the effect of systemic therapies, as well as the prevention of complications affecting other organs. Given the possible increase in blood viscosity and decrease in circulation in paraproteinemia, our purpose in this study was to evaluate retinal blood flow using OCT angiography in patients with monoclonal gammopathy.

## 2. Materials and Methods

In this cross-sectional study, 44 eyes of 27 patients with monoclonal gammopathy (11 male and 16 female, mean age: 63.59 ± 9.33 years) were recruited from the Department of Hematology and Stem Cell-Transplantation of the South-Pest Central Hospital, National Institute for Hematology and Infectious Diseases (Budapest, Hungary) and the 3rd Department of Internal Medicine, Semmelweis University (Budapest, Hungary). The patients were diagnosed and treated between 1997 and 2020. The study was conducted according to the Declaration of Helsinki, concerning the relevant national and local requirements, and was approved by the National Drug Agency’s Ethical Review Board for Human Research (OGYÉI/50115/2018). All subjects gave their written informed consent. Regarding the hematological diagnosis, MGUS was diagnosed in 3 (11.11%), multiple myeloma in 21 (77.77%), smoldering myeloma in 1 (3.7%), and amyloidosis in 2 (7.4%) patients. As a control group, 62 eyes of 36 age-matched healthy individuals (11 male and 25 female, mean age: 58.01 ± 11.46 years) were involved in the study.

Patients with diabetes and those with any history of intraocular surgery, previous intravitreal anti-VEGF injection or laser treatment, or other ophthalmological diseases—such as glaucoma, age-related macular degeneration, vitreomacular disease, or refractive errors > 6 diopters—were excluded from the study. All the patients underwent OCT angiography imaging using the Optovue AngioVue OCT angiography system (2017.1 software version, phase 7.0 update) with an SSADA (split-spectrum amplitude-decorrelation angiography) software algorithm, by which 3 × 3 mm OCT angiograms were performed, centered at the macula. The superficial retinal vessel density (SVD) and deep retinal vessel density (DVD) were evaluated in the central 3 × 3 mm and parafoveal area, and the size of the foveal avascular zone (FAZ) at the level of the superficial capillary plexus and central retinal thickness (CRT) were measured using the built-in automated AngioAnalytics software of the OptoVue system. The parafoveal area was defined as a ring-shaped region with an inner radius of 1.5 mm from the center of the fovea, excluding a central foveal 0.5 mm radius area. Scans with segmentation errors at the superficial or deep vascular plexus level and images with artifacts (double vessel pattern, dark areas from blink, white line artifacts, and vessel discontinuities induced by microsaccades) were excluded from the study. Only OCT angiograms with a scan quality (SQ) index of 6 or above were accepted for further analysis—as recommended by OptoVue as a threshold for acceptable image quality.

### Statistical Analysis

The statistical analysis was performed with SPSS software (version 27.0, IBM, Armonk, NY, USA). The effect of the image quality on the OCTA parameters was assessed with a multivariable regression analysis using generalized estimating equation (GEE) models. This test enabled adjustments to be made for the within-subject correlation of the parameters (right vs. left eye) by taking into account the between-eye correlations. Moreover, the inclusion of scan quality as a covariate in GEE models permits one to simultaneously control for its effect on the dependent variables, providing valid *p* values for group comparisons.

## 3. Results

There were no significant differences in terms of age and gender between the monoclonal gammopathy and healthy control groups (*p* > 0.05).

**Figure 1:**
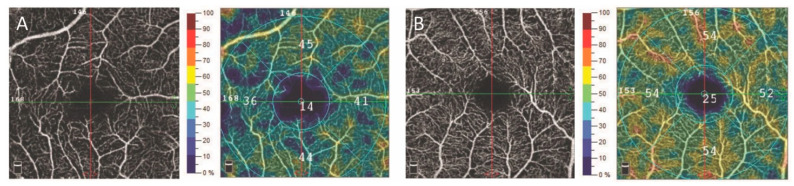
En-face OCT angiogram and retinal vessel density map of the superficial retinal vascular plexus measured in the 3 × 3 mm macular using by OptoVue AngioVue OCT angiography in a patient with monoclonal gammopathy (**A**), and a healthy subject (**B**). OCT: optical coherence tomography.

The values of the SQ ranged from 6 to 9, with an overall mean of 7.05 ± 1.12 in the eyes with monoclonal gammopathy and 7.83 ± 1.03 in the normal subjects (*p* = 0.001). The superficial retinal vessel density values showed a significant positive correlation with the SQ values in both groups (beta: 0.9, 95%CI: 0.19–1.61; *p* = 0.01), but there was no significant correlation between the SQ and other OCTA parameters (*p* > 0.05 for all the parameters). The superficial retinal vessel density was significantly decreased in the central 3 × 3 mm macular area in the patients with monoclonal gammopathy compared to the healthy individuals, after controlling for the effect of signal quality in a multivariable regression model (Figure 1). However, no significant difference was found regarding the superficial retinal vessel density in the parafoveal zone, the deep retinal vessel density both in the 3 × 3 mm macular and parafoveal zone, the foveal avascular zone (FAZ) area, and the central retinal thickness (CRT) values between the two groups (Table 1).

## 4. Discussion

Monoclonal gammopathy may have several ocular manifestations, affecting most ocular tissues, and these are not uncommonly the first symptom of the disease. In a previous paper, our study group evaluated the ocular signs and ocular comorbidities in 80 patients with monoclonal gammopathy. According to our results, ocular surface disease and cataracts are more common in subjects with monoclonal gammopathy than in age-matched controls [7]. We also demonstrated that increased corneal light scattering in the central 10 mm annular zone and increased keratocyte hyperreflectivity, evaluated using Pentacam and in vivo confocal microscopy, respectively, may give rise to a suspicion of monoclonal gammopathy [8]. Nevertheless, in contrast to the anterior segment involvement in this population, we have much less knowledge about the involvement of the posterior segment. Due to the alterations in the systemic circulation in monoclonal gammopathy, quantitative measurements of retinal blood flow using OCT angiography could offer an easy and fast method for detecting even subtle circulatory changes.

In monoclonal gammopathy, owing to the high level of paraproteins, major hemorheological changes occur in the circulation that may be as serious as hyperviscosity syndrome. Hypergammaglobulinemia—especially Waldenström macroglobulinemia—is the most frequent cause of hyperviscosity syndrome. An increase in blood viscosity can result from either erythrocyte aggregation or an increased plasma viscosity. These changes depend on the plasma concentration, as well as the molecular size of the paraprotein, with a threshold for the onset of hyperviscosity for IgG of >15 g/dL, for IgA of >1 g/dL, and for IgM of >3 g/dL. Accordingly, symptomatic hyperviscosity in Waldenström macroglobulinemia is more common, affecting 10–30% of patients, than IgG myeloma (2–6%) is [17]. Nevertheless, after taking into account the above exclusion criteria, patients with Waldenström macroglobulinemia were not included in our present study; only MGUS, multiple myeloma, smoldering myeloma, and amyloidosis subjects could be analyzed. In monoclonal gammopathy, an increased blood viscosity causes sluggish blood flow and decreased microvascular circulation, leading to the hypoperfusion of tissues. The classic triad of hyperviscosity syndrome consists of mucosal or skin bleeding, neurological deficits, and visual disturbances [18]. Ophthalmic findings include venous dilation, flame hemorrhages, papilledema, exudates, and microaneurysm formation. The most severe ophthalmic manifestation of hyperviscosity syndrome is central retinal vein or artery occlusion, which can result in irreversible vision loss [5].

Physiologically, blood flow is influenced by blood velocity, vessel diameter, and whole-blood viscosity, which is determined by hematocrit, plasma viscosity, red cell aggregation, and deformability [19]. A previous study conducted by Uggla et al. examined the hemorheological patterns of 87 patients with monoclonal gammopathy, including MM (n = 52), MGUS (n = 30), and WM (n = 5). Of the MG patients, 71% had a plasma viscosity above the reference limit and 40% were above the whole-blood viscosity limit [20]. Another study group observed significant increases in whole-blood viscosity and plasma viscosity, as well as a marked decrease in erythrocyte deformability in MGUS patients compared to controls [19].

OCT angiography is a new imaging technique based on optical coherence tomography that can visualize the functional blood vessels in the eye by detecting the variation in the OCT signal caused by moving particles. During image acquisition, repeated OCT scans are performed at the same location of the retina, and the backscattered OCT signals from the moving red blood cells in the vessels are differentiated from the backscattered OCT signals from static structural tissue [21]. Commercially available OCTA devices are typically used to create en-face images with red blood cell movement occurring within a range from 0.3 to 3 mm/s. As human retinal capillary flow speeds have been estimated to be in the range of 0.4–3.0 mm/s, this suggests that SSADA is well suited for detailed angiography down to the capillary level [22].

After performing OCT angiography studies examining various ophthalmological diseases, different systemic disorders are also increasingly becoming the focus of the research into this novel imaging technique. The major advantage offered by OCT angiography is that it is an excellent non-invasive tool for screening and monitoring vascular diseases, allowing for closer follow-ups of these patients. Several studies have been published using OCT angiography in systemic diseases, such as hypertension, diabetes mellitus, coronary artery disease, carotid artery stenosis, preeclampsia, kidney disease, and hematological disorders. Previous reports have illustrated that OCT angiography can detect retinal microvascular alterations in systemic hypertension and high cardiovascular risks. Therefore, monitoring these changes before irreversible end-organ damage occurs could prevent life-threatening hypertensive-related complications [23,24]. Since OCTA has become available, numerous studies have reported changes in the retinal microvasculature of patients with diabetes. Our study group demonstrated that, regardless of the duration of diabetes, a reduced retinal vessel density was linked to a significantly increased rate of diabetic retinopathy, and as a consequence, OCTA metrics may serve as prognostic biomarkers for the prediction of early-onset diabetic retinopathy [25]. We also analyzed the retinal circulation of patients with severe carotid artery stenosis using OCT angiography and found that carotid surgery resulted in a significant improvement in retinal blood flow, both ipsi- and contralaterally, independent of systemic factors. This finding corroborates those of Lee et al. [26,27]. Accordingly, OCT angiography could also help in the assessment of cerebral circulation changes related to carotid artery stenosis. Another recent study described that subjects with end-stage renal disease receiving hemodyalisis showed a decreased flow density in the superficial capillary and choriocapillary plexus in OCTA imaging, and that flow density was negatively correlated with hemodyalisis treatment. In contrast, the deep capillary plexus appeared to be more resilient towards hemodynamic changes caused by hemodyalisis treatment [28]. Additionally, OCT angiography may also be beneficial for neurological research to advance our understanding of the pathophysiology of multiple sclerosis, Alzheimer’s disease, and several optic neuropathies [29]. According to the results published in the literature, OCT angiography provides extremely useful information concerning the blood flow both in the retina and in the whole body. There is an increasing number of studies that are devoted to expanding the usability of OCT angiography in the management of systemic diseases, which previously did not include assessments of the retinal vasculature [30].

Currently, there have only been a few reported studies on OCT angiographic evaluations of the retinal circulation in patients with monoclonal gammopathy. A previous report described a case of hyperviscosity retinopathy due to Waldenström macroglobulinemia, with no abnormalities on OCT angiography [14]. However, in a study conducted by Li et al., OCT angiography showed characteristic changes in both the retinal and choroidal vasculatures in a patient with Waldenström macroglobulinemia retinopathy, as macula edema presented as petaloid cysts in the outer plexiform layer, the capillary network of the macula was blurred and irregular, and there were augmented choroidal large blood vessels [15]. Considering the exclusion criteria, OCTA measurements of patients with Waldenström macroglobulinemia were not included in our study for further statistical analysis.

In the present study, the superficial retinal vessel density measured using OCT angiography was significantly decreased in the eyes of the patients with monoclonal gammopathy compared to the controls. Nevertheless, we found no significant differences regarding the other OCTA parameters—the deep retinal vessel density, foveal avascular zone area, and central retinal thickness—between the two groups. In line with our findings, Dursun et al. detected a decreased vessel density in the superficial and deep macular retinal areas and papillary and peripapillary regions measured with OCT angiography, suggesting a decreased blood flow and possible ischemia in patients with multiple myeloma. However, they did not take into account the effect of image quality on the OCTA parameters, which is inevitable for the appropriate assessment of the values, as well as for longitudinal patient follow-up [16,31,32].

Consistent with our prior findings, in the current study, the image quality (SQ index) was positively correlated with the superficial retinal vessel density values in both groups, demonstrating that the OCTA parameters are significantly different in scans with a lower image quality compared to those with better quality [31,32].

Furthermore, we classified the patients with monoclonal gammopathy according to the International Staging System (ISS) and Revised ISS, based on their serum levels of albumin, β2 microglobulin, and lactate dehydrogenase (LDH), and the presence of high-risk chromosomal abnormalities (CA) [33,34]. In our study, 16 patients were assigned to ISS stage I (59%), 3 patients to ISS stage II (11%), and 8 patients to ISS stage III (30%), whereas 15 patients were assigned to R-ISS stage I (56%), 10 patients to R-ISS stage II (37%), and 2 patients to R-ISS stage III (7%). According to the ISS and Revised ISS, we found no significant difference regarding the OCTA parameters between the different-stage groups. Nevertheless, given that the blood tests of the patients with monoclonal gammopathy and the OCT angiographic examinations were not performed simultaneously, in our study, these data were not included in the manuscript for further statistical analysis, owing to a possible change in blood flow occurring between the two visits.

Monoclonal gammopathy is associated with hemorheological abnormalities—including an increase in blood viscosity—that cause a decreased blood flow. OCT angiography is a novel technology that can detect very mild capillary circulation disorders, which can be an early predictor of hyperviscosity syndrome in these subjects. A better identification of patients with decreased circulation could prevent the different complications of hyperviscosity syndrome, such as bleeding disorders, vision-threatening complications, and neurological deficits. Moreover, the earlier detection of microcirculatory disorders may allow closer monitoring for the further progression of the condition.

We acknowledge that our study has some limitations, including its small sample size and the cross-sectional nature of the work, with a lack of follow-up to define accuracy measures in the patients with monoclonal gammopathy. In addition, it would have been noteworthy to analyze the association between the blood parameters, visual acuity, and OCTA parameters simultaneously. In the future, larger prospective studies will be needed to confirm our results, in order to highlight the role of OCT angiography in disease monitoring and therapeutic follow-up.

## 5. Conclusions

In summary, a decreased superficial retinal vessel density measured using OCT angiography in patients with monoclonal gammopathy suggests the presence of a sluggish blood flow, reduced capillary circulation, and consequent tissue hypoperfusion due to an increased blood viscosity. An OCT angiographic examination of the retinal blood flow in monoclonal gammopathy—taking into account the influence of the image quality on the OCTA parameters—can be a sensitive method for the non-invasive detection and follow-up of early microcirculatory disorders caused by hyperviscosity.

## Figures and Tables

**Table 1:** OCTA parameters in patients with monoclonal gammopathy and healthy individuals.

	Monoclonal Gammopathy (*n* = 44)	Healthy Controls (*n* = 62)	*p*
VD SVP—3 × 3 mm central macular area (%)	44.54 ± 3.22	46.62 ± 2.84	0.04
VD DVP—3 × 3 mm central macular area (%)	48.59 ± 3.23	49.76 ± 3.99	0.77
VD SVP—parafoveal area (%)	47.54 ± 3.60	49.57 ± 2.95	0.08
VD DVP—parafoveal area (%)	51.13 ± 3.24	52.00 ± 3.99	0.93
FAZ (mm^2^)	0.293	0.289	0.91
CRT (µm)	263.73	254.95	0.22

OCTA: optical coherence tomography angiography, VD: vessel density, SVP: superficial vascular plexus, DVP: deep vascular plexus, FAZ: foveal avascular zone, CRT: central retinal thickness, and p: the difference between the two groups after controlling for scan quality in multivariable models.

## Data Availability

Data will be available on request from the corresponding author.
